# HAS THE RACIAL/ETHNIC DIGITAL DIVIDE AMONG OLDER COMMUNITY DWELLERS DEEPENED OR IMPROVED IN THE 2010S?

**DOI:** 10.1093/geroni/igad104.2205

**Published:** 2023-12-21

**Authors:** Xiayu Chen, J Zak Peet, Danan Gu, Kun Wang

**Affiliations:** University of Illinois at Urbana-Champaign, Champaign, Illinois, United States; Binghamton University, Binghamton, New York, United States; The United Nations, New York City, New York, United States; Binghamton University, State University of New York, Binghamton, New York, United States

## Abstract

After the rapid societal and technological changes that occurred during the 2010s, it is unclear whether the racial/ethnic digital divide has deepened or improved. Guided by critical race theory, this study aims to examine trends of the racial/ethnic effect on the first- and second-level digital divide and how race/ethnicity interacts with gender and education. Data from three rounds (2011, 2015, and 2019) of the National Health and Ageing Trend Study were used in this study. Older community dwellers with normal cognition were included (2011: n = 5214; 2015: n = 5543; and 2019: n = 3242). The first-level digital divide was measured by Internet access. The second-level digital divide was measured by the usage of six online activities. Using weighted multiple logistic regressions, we found that race/ethnicity became not significantly associated with the first-level digital divide in 2019 but was consistently associated with the second-level divide. Neither gender nor education moderated the first-level digital divide association across the three years. For the second-level digital divide, education moderated the racial/ethnic digital divide on online grocery shopping and banking in 2011 and 2015 but not in 2019; by contrast, education’s moderation effects on buying prescriptions, contacting health providers, and getting health information online were significant in 2015 and 2019. No general trends were found for the moderation effect of gender on the second-level digital divide. Racial/ethnic gaps in the first-level but not the second-level digital divide were alleviated. More interventions should be provided for racial/ethnic minorities, especially those with low education.

